# IL-18-Mediated Tumor Immune Evasion

**DOI:** 10.3390/cimb48020202

**Published:** 2026-02-12

**Authors:** Shuai Li, Chenxia Gao, Hongyu Zhao, Didi Wang, Shuang Liu

**Affiliations:** School of Basic Medicine, Jiamusi University, Jiamusi 154007, China; 15686061872@163.com (S.L.); g15633612105@163.com (C.G.); 13260663259@163.com (H.Z.)

**Keywords:** IL-18, tumor immunity, immune evasion, IL-18BP, tumor microenvironment

## Abstract

Immune response evasion is one of the hallmark features of cancer, which is not only the basis for cancer progression and metastasis but also affects the clinical management of cancer. Tumor immune evasion is mainly attributed to the dynamic and immunosuppressive tumor microenvironment (TME), which is regulated by a complex system including immunosuppressive cells and cytokines. Interleukin-18 (IL-18) is an important cytokine that plays a multifaceted role in immune system regulation, and its function is strictly regulated by the natural antagonist IL-18 binding protein (IL-18BP). IL-18 exhibits context-dependent immunoregulatory characteristics (acting as a “context resistor”) during tumor occurrence and progression, which is closely related to cancer type, stage, and the signaling network of the tumor microenvironment. The multifaceted functions of IL-18 have been utilized in cancer treatment to reduce the phenomenon of immune escape of tumors. With the latest advancements in cancer research related to IL-18, it is necessary to integrate the latest research findings to deepen the understanding of the mechanism of tumor immune escape and promote the improvement of cancer treatment levels. This review will systematically elaborate on the action mode, core regulatory mechanism and key signaling pathways of IL-18 in tumor immune evasion, analyze the heterogeneity patterns associated with its context-dependent effects, comprehensively sort out the core obstacles in clinical translation, and at the same time, envision new precision treatment strategies based on IL-18 regulation.

## 1. Introduction

**Figure 1:**
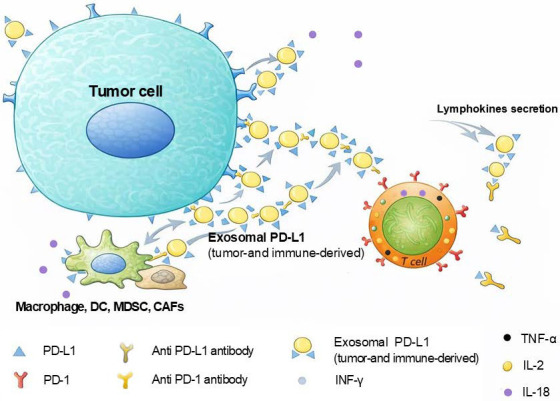
Tumor immune evasion.

Tumor immune evasion, as a critical component in cancer development and metastasis, has become a pivotal focus in oncology research [1]. Within the tumor microenvironment (TME), cancer cells employ multiple mechanisms to evade immune surveillance, thereby promoting their survival and proliferation. This process involves not only alterations in tumor cell characteristics but also complicated regulation of immunosuppressive cells and cytokines within TME [2] (Figure 1). For instance, the accumulation of regulatory T cells (Tregs) and myeloid-derived suppressor cells (MDSCs) significantly impairs effector T cell function while fostering immune tolerance in tumor cell [3]. Additionally, tumor cells can suppress immune recognition and cytotoxicity by downregulating major histocompatibility complex (MHC) molecule expression or upregulating immune checkpoint molecules such as programmed death ligand-1 (PD-L1), thereby exacerbating immune evasion [4]. This immunosuppressive system is regulated by chemokines and cytokines.

In recent years, with in-depth research, the complexity of IL-18’s role in tumors has gradually emerged. The simplistic early view of “antitumor at low concentrations and protumor at high concentrations” can no longer explain the heterogeneous effects of IL-18 across different tumor types. For instance, IL-18 often exhibits antitumor activity in solid tumors such as colorectal cancer, but may promote tumor progression in glioma and pancreatic cancer. This discrepancy is not solely determined by concentration but results from the combined action of multiple factors including cellular source, IL-18BP expression level, receptor density, and local cytokine environment. Therefore, this review proposes the concept of “contextual resistor” to more accurately describe IL-18’s immunomodulatory characteristics.

Interleukin-18 (IL-18), a crucial pro-inflammatory cytokine, has garnered significant attention in recent immunomodulatory research. The balance of its biological functions depends on the interaction with the natural antagonist IL-18 binding protein (IL-18BP) [5]. It is primarily produced by immune cells such as macrophages and dendritic cells (DC), IL-18 performs biological functions including inducing interferon-γ (IFN-γ) production, enhancing natural killer (NK) cell cytotoxicity, and promoting T cell proliferation and differentiation [6,7]. In recent years, with in-depth research, the complexity of IL-18’s role in tumors has gradually emerged. IL-18 plays a context-dependent immunomodulatory role in tumor immunity: its effect is determined by key factors such as local concentration, cell origin, IL-18:IL-18BP ratio, receptor density, and exposure mode (acute vs. chronic) [8]. Specifically, under certain conditions, IL-18 can enhance the Th1-type immune response by activating NK cells and CD8+ T cells, thereby inhibiting tumor growth; while in other situations (such as high expression of IL-18BP or chronic exposure), it may promote tumor progression by inducing cancer cells to express immune escape-related molecules or regulating the immunosuppressive network in the TME [9,10]. Moreover, IL-18 initiates signal transduction by binding to the IL-18 receptor (IL-18R), activating downstream pathways such as NF-κB and MAPK, and regulating the expression and release of various inflammatory factors. These mechanisms are all involved in the regulation of IL-18 on cancer progression [11]. A large amount of evidence indicates that IL-18 affects the immunogenicity and sensitivity of tumors by regulating NK cells and T cells in the TME. IL-18 interacts with other cytokines/signal pathways to jointly shape an immunosuppressive TME. The synergistic effect of IL-18 and IL-12 can enhance Th1-type immune responses, while its binding to vascular endothelial growth factor (VEGF) may promote tumor angiogenesis and invasiveness [12,13]. By activating the VEGF signaling pathway, IL-18 can enhance the proliferation and migration ability of vascular endothelial cells, promote tumor vascular formation, and thereby accelerate the spread of cancer cells [14,15]. Additionally, therapies based on IL-18 (such as immune checkpoint inhibitors combined with immunotherapy, chimeric antigen receptor T-cell (CAR-T) therapy) have shown significant antitumor effects in some tumor models, but there are also potential risks of translational application that require careful evaluation.

A review published in *The International Journal of Molecular Sciences* in 2024 systematically summarized the multifaceted functions of IL-18, its role in cancer, and treatment strategies, providing a comprehensive theoretical framework for research in this field [16,17,18,19]. Based on this, this review further focused on the specific molecular mechanisms by which IL-18 mediates tumor immune escape, supplemented with the latest data from CAR-T cell therapy research, clearly distinguishing causal relationships from correlations among the mechanisms, improving the research progress of the IL-18BP-related regulatory network, and proposing the “IL-18 Threshold and Contextual Regulation Model” to integrate key regulatory factors, providing new experimental basis and ideas for developing more precise targeted treatment strategies.

## 2. Biological Properties of IL-18

### 2.1. IL-18 and Its Natural Antagonist IL-18BP

IL-18 is expressed in various tissues and cells, with mononuclear/macrophages being its primary source [20]. IL-18 mRNA is most abundant in human pancreatic, renal, and skeletal muscle tissues, while also showing varying levels of expression in liver, lung tissues, epidermal keratinocytes, adrenal glands, and intestinal epithelial cells [21]. Notably, despite its widespread distribution across many tissues, IL-18 shows minimal expression in T and B cells, suggesting its functions may primarily rely on innate immunity rather than adaptive immunity. Additionally, IL-18 expression is regulated by diverse factors. Pathogen-associated molecular pattern (PAMPs) and damage-associated molecular patterns (DAMPs) can both induce its upregulation, thereby participating in host immune defense responses [22]. These characteristics position IL-18 as a crucial bridge connecting innate and adaptive immunity, playing a pivotal role in tumor immune evasion.

IL-18 is a cytokine with multifunctional immune regulatory roles [23,24,25]. Its molecular structure exhibits a distinctive β-triclane spatial configuration, which closely resembles that of IL-1 family members. Specifically, IL-18 consists of 12 β-sheet chains forming a compact and stable three-dimensional structure, providing essential structural foundations for receptor binding and signal transduction. Additionally, the IL-18 precursor molecule requires cleavage by IL-1β-converting enzyme at the Asp-X position to remove a 36-amino acid pre-sequence before becoming the active mature form [26]. This processing mechanism not only determines IL-18’s functional state but also reflects the complication of its intracellular signaling pathways. Structurally, IL-18’s spatial conformation enables efficient binding to IL-18R on cell membranes, thereby initiating downstream signaling pathways and further regulating immune responses.

IL-18BP, first identified and reported in 1999, is not merely a passive antagonist but a secreted active immune checkpoint [27,28]. This molecule binds to both wild-type IL-18 and decoy-resistant IL-18 variants with nanomolar-level high affinity, exerting a dominant inhibitory effect on the IL-18 signaling pathway by blocking the formation of the IL-18/IL-18Rα/IL-18Rβ ternary complex; even at low concentrations, it can effectively neutralize the biological activity of IL-18. In cancer, IL-18BP shows tumor-type specific expression and is mainly secreted by tumor cells, regulatory T cells, MDSCs and other cell types [15,29], with its expression level closely correlated with the degree of tumor immunosuppression and poor patient prognosis. For example, IL-18BP is significantly upregulated in tumor tissues or serum from patients with colorectal cancer, esophageal cancer and brain tumors, which facilitates tumor immune evasion by neutralizing the antitumor activity of IL-18 [30,31,32]. Notably, the balanced regulation of IL-18 and IL-18BP is essential for maintaining immune homeostasis, and the imbalance in their expression plays a pivotal role in tumor immune evasion.

### 2.2. IL-18 Receptor and Signal Transduction Pathway

**Figure 2:**
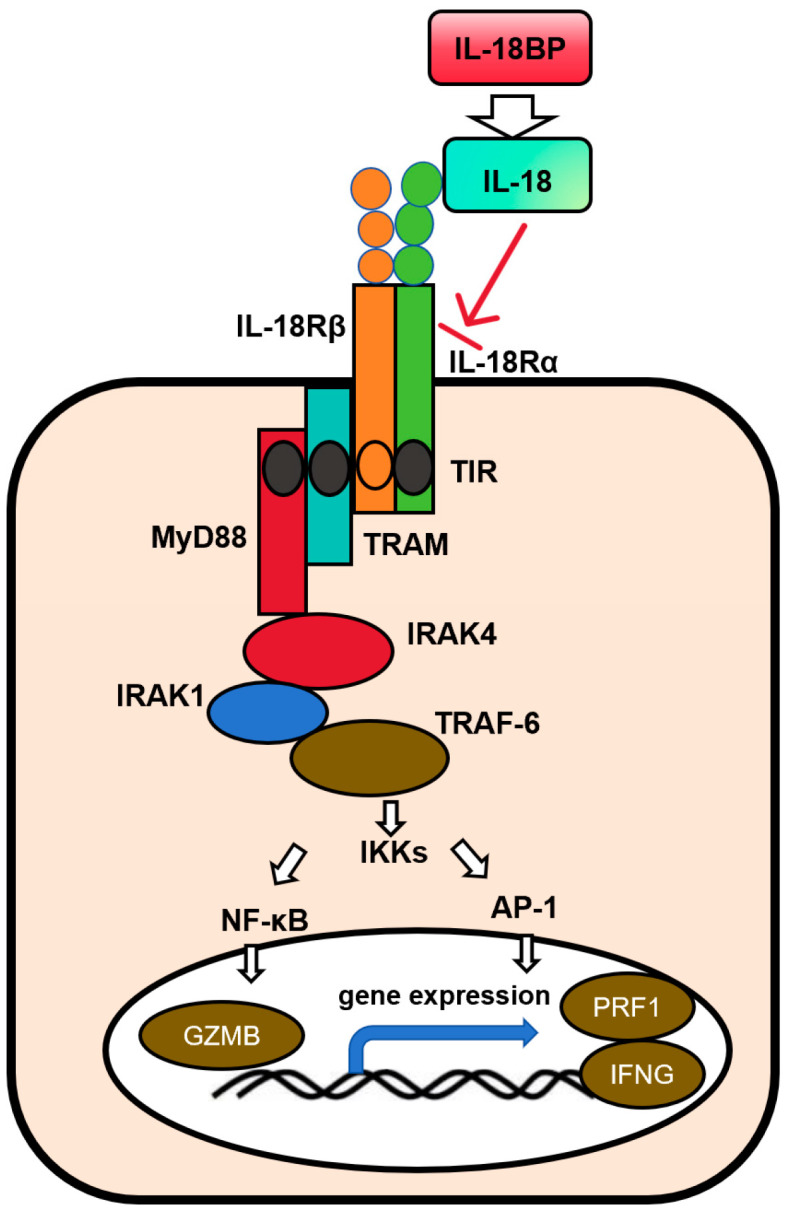
IL-18 signal transduction.

IL-18 is a member of the IL-1 family. Its precursor form is biologically inactive and requires cleavage by caspase-1 to form the mature active molecule. IL-18 initiates signal transduction by binding to the interleukin-18 receptor (IL-18R) on the cell membrane. IL-18R is a heterodimer composed of IL-18Rα (the ligand-binding subunit) and IL-18Rβ (the signaling subunit), where IL-18Rα is responsible for the specific binding of IL-18, and IL-18Rβ is involved in the formation of an active signaling complex [33]. Upon binding, IL-18R recruits the adaptor protein MyD88, which in turn triggers the phosphorylation of IL-1 receptor-associated kinases (IRAKs) and the recruitment of TRAF6 [34]. This ultimately activates the two transcription factors NF-κB and AP-1, promoting the secretion of pro-inflammatory cytokines including IL-1β, IL-6, IL-8, as well as granulocyte-macrophage colony-stimulating factor (GM-CSF) [35]. In NK cells, IL-18 can also activate the mitogen-activated protein kinase (MAPK) pathway, which includes the ERK, JNK, and p38 MAPK subtypes [27,28]. This activation coordinately regulates the transcription and translation of interferon-γ (IFN-γ), while enhancing the expression of killer receptors on the surface of NK cells and the release of cytotoxic molecules such as perforin and granzyme, thereby significantly boosting their antitumor effector functions (Figure 2). It is important to clarify that IL-18 signal transduction is not mediated by Toll-like receptors (TLRs), and this mechanism is highly conserved across different cell types. Meanwhile, the dependence on MyD88 varies among cell types (e.g., MyD88 deficiency in NK cells significantly impairs the activating effect of IL-18).

## 3. IL-18 and Tumor Immune-Evasion-Related Mechanisms

### 3.1. Mechanisms of Tumor Immune Evasion

TME constitutes a complex ecosystem comprising tumor cells, immune cells, vascular endothelial cells, and various soluble factors, playing a pivotal role in tumor immune evasion. The immune cell population is enriched with immunosuppressive cells such as Tregs, MDSCs, and tumor-associated macrophages (TAMs) [36,37,38]. These cells significantly impair the antitumor efficacy of effector T cells and NK cells by secreting immunosuppressive factors like IL-10, TGF-β, and IL-18BP. Notably, IL-18BP, acting as a natural antagonist to IL-18, competitively binds to IL-18, thereby blocking its activation of NK cells and T cells, which promotes tumor immune evasion [6]. Additionally, inflammasomes in the TME have been confirmed to participate in immune evasion processes. For instance, NLRP3 inflammasomes activate caspase-1 to induce the secretion of IL-18 and IL-1β [39,40,41]. However, these cytokines may undergo reprogramming, in the context of tumors which can be largely categorized into stromal cells and tumor cells, to support tumor growth and metastasis. At the same time, tumor cells and immune suppressive cells can secrete IL-18BP, further regulating the functional balance of IL-18.

Tumor cells employ multiple mechanisms to evade immune system recognition and attack, with the expression of immune checkpoint molecules and reduced antigen presentation being the two most critical approaches. On one hand, tumor cells overexpress immune checkpoint molecules such as programmed death ligand-1 (PD-L1) and cytotoxic T lymphocyte-associated antigen-4 (CTLA-4) on their surfaces, which bind to corresponding receptors on immune cell surfaces, thereby inhibiting T-cell activation and proliferation [42]. On the other hand, tumor cells significantly reduce their immunogenicity by downregulating MHC molecule expression or disrupting antigen processing and presentation mechanisms, making it difficult for effector T cells to recognize and eliminate them [43,44]. Additionally, tumor cells can further weaken immune cell antitumor activity by secreting immunosuppressive factors like IL-18BP and TGF-β [29]. Notably, IL-18 may enhance immune evasion capabilities by inducing tumor cells to express immune checkpoint molecules such as PD-L1 in the TME. These mechanisms not only reveal the complication of tumor cells’ immune evasion strategies but also provide theoretical foundations for developing novel therapeutic approaches based on IL-18.

### 3.2. Mechanism of IL-18-Mediated Tumor Immune Evasion

#### 3.2.1. Regulation of Immune Cell Function in TME

Immune cells play a crucial role in antitumor immune responses in the TME. IL-18 affects various aspects of TME by regulating immune cell recruitment, NK and CD8+ T cell activation, as well as contributing to immune tolerance via facilitating the actions of Treg, MDSC, and TAM. IL-18BP participates in this complex regulatory network by regulating the biological activity of IL-18.

IL-18 enhances NK cell activity by significantly increasing the cytotoxicity derived from NK cells and promoting the production of IFN-γ. In a melanoma study, IL-18 treatment increased the tumor killing activity of NK cells and inhibited tumor growth [18]. In addition, IL-18 exerts its antitumor effect by enhancing antibody-dependent cell-mediated cytotoxicity (ADCC). The ADCC mechanism involves the binding of antibodies to tumor cell surface antigens, followed by activation of NK cells by recognizing the Fc segment of antibodies through their surface Fc receptors [45]. However, immune suppressive cells in the TME, such as Tregs and MDSCs, can secrete IL-18BP. This protein acts as a natural antagonist of IL-18 and can competitively bind with IL-18, thereby blocking its activation of NK cells and T cells and indirectly weakening its antitumor activity [6,29]. Experimental data shows that the use of IL-18BP inhibitors can restore the biological activity of IL-18, thereby enhancing the ability of NK cells to kill tumors and significantly inhibiting tumor growth [8]. In addition, IL-18 induces NK cells to express programmed death receptor-1 (PD-1) on their surface, allowing them to bind to PD-L1 on the tumor cell membrane to inhibit its activity [46]. These results confirmed that blocking the PD-1/PD-L1 pathway significantly enhances the antitumor effect of IL-18. This phenomenon indicates that IL-18 has multiple regulatory modes in regulating NK cell function.

Beyond its effects on NK cells, IL-18 exerts a significant bidirectional regulatory role in T cell function. Under physiological conditions or at low concentrations, this cytokine can promote T cell proliferation and activation, thereby enhancing their antitumor capacity [47]. IL-18 enhances the ability of T cells to recognize and eliminate cancer cells by upregulating the surface receptor NKG2D [48]; in vitro experiments have confirmed that T cells with elevated NKG2D expression exhibit a markedly improved tumor-killing efficiency [49]. However, in the complex context of the TME, IL-18 can drive T cell exhaustion through a specific molecular pathway, thereby impairing antitumor immune responses. This process involves three key steps: First, IL-18 secreted by tumor cells and TAMs binds to the IL-18Rα/β heterodimeric receptor on the surface of effector T cells, triggering intracellular signal cascades; it then upregulates the gene transcription and protein expression of inhibitory receptors such as PD-1 and Tim-3 via the phosphorylation of STAT3 and activation of the NF-κB pathway [50]. Second, the continuously overexpressed inhibitory receptors bind to their ligands (e.g., PD-L1, Gal-9), initiating immunosuppressive signals within T cells; this leads to reduced secretion of cytokines such as IFN-γ and TNF-α by effector T cells, a marked decrease in the cytotoxic effects of granzyme B and perforin, and the gradual development of an irreversible exhausted phenotype [50]. Third, exhausted T cells recruit and activate Tregs, and reshape the immunosuppressive TME by secreting inhibitory cytokines including IL-10 and TGF-β; these cells not only lose their intrinsic antitumor capacity but also suppress the immune functions of NK cells and DC, ultimately impairing the host antitumor immune response at both the cell-autonomous and microenvironmental regulatory levels, thus creating favorable conditions for tumor immune evasion [51]. Further studies have shown that Tregs may directly inhibit IL-18 function by secreting IL-10 and TGF-β, and indirectly modulate IL-18 activity by promoting the secretion of IL-18BP, thereby exacerbating immune evasion in the TME [52]. Notably, blocking Treg function can restore the antitumor effects of IL-18 [52]. Collectively, these findings indicate that IL-18 plays a crucial dual regulatory role in modulating T cell function.

IL-18 demonstrates significant regulatory effects on T cell proliferation, differentiation, and function. By synergizing with IL-12, IL-18 promotes Th1 cell differentiation and induces IFN-γ production, thereby enhancing Th1-type immune responses [53]. This mechanism not only activates cytotoxic T cells (CTL) but also effectively eliminates cancerous and viral-infected cells. However, it is noteworthy that IL-18 has relatively limited effects on Th2 cytokines. For instance, human IL-18 reduces IL-10 production in jack bean protein A stimulated peripheral blood mononuclear cell (PBMC), yet shows no significant impact on IL-4 production [54]. This selective regulation suggests that IL-18’s role in tumor immune evasion may be closely related to its modulation of the Th1/Th2 balance. IL-18BP can affect the establishment of this balance by regulating the effective concentration of IL-18.

Furthermore, IL-18 exerts significant effects on the functions of other immune cells such as macrophages and dendritic cells. In macrophages, IL-18 activates the MAPK cascade to induce IFN-γ production, thereby enhancing their phagocytic and cytotoxic capabilities [55]. For DC, IL-18 promotes their maturation and improves antigen presentation efficiency, thereby strengthening adaptive immune responses. However, these immune cells’ functions are often suppressed by diverse factors in the TME [56]. For instance, tumor-derived IL-18 may further weaken antitumor immune responses by inducing the recruitment of MDSCs [57]. Therefore, the regulation of immune cell functions by IL-18 constitutes a multi-layered complicated process, with specific effects depending on tumor type and the specific conditions of the microenvironment.

#### 3.2.2. Effects on the Intrinsic Characteristics of Tumor Cells

Beyond regulating immune cell functions, IL-18 directly influences tumor cell characteristics through multiple mechanisms, thereby promoting their proliferation, invasion, and metastasis while inducing the expression of immune evasion-related molecules. IL-18BP can indirectly regulate these processes by neutralizing the activity of IL-18. First, IL-18 enhances tumor cell proliferation by modulating the PI3K/AKT signaling pathway [53]. Studies have shown that IL-18 upregulates VEGF expression, a process dependent on activation of the PI3K/AKT signaling pathway [58]. VEGF, as a key angiogenic factor, plays a central role in tumor angiogenesis. Its high expression can promote the nutrient supply and metabolic waste excretion of tumor tissue, providing necessary conditions for tumor proliferation. It has been confirmed that high expression of VEGF is associated with tumor progression and poor prognosis in cancer [15]. Additionally, IL-18 further promotes tumor cell proliferation and survival by inducing the expression of MMP-2 [59]. This regulation of proliferative signaling pathways establishes IL-18 as a crucial player in tumor development and progression. IL-18BP can weaken these protumor effects by inhibiting the activity of IL-18.

Secondly, IL-18 significantly influences the invasive and metastatic capabilities of tumor cells. Matrix metalloproteinases (MMPs), enzymes that degrade extracellular matrices, have their expression levels closely linked to tumor cell invasiveness and metastasis potential [60]. Research indicates that IL-18 enhances tumor cell invasion by activating the NF-κB signaling pathway, which induces the expression of MMP-2 and MMP-9 [61]. Additionally, IL-18 promotes the epithelial-mesenchymal transition (EMT) process in tumor cells, further empowering their invasive and metastatic potential [62]. This regulation of invasion and metastasis-related molecules demonstrates that IL-18’s role in tumor progression extends beyond immune modulation to involve malignant behaviors inherent in tumor cells. IL-18BP may inhibit tumor invasion and metastasis by blocking these effects of IL-18.

Finally, IL-18 can induce tumor cells to express immune checkpoint molecules and immunosuppressive molecules, thereby evading immune system attacks. For example, IL-18 enhances the expression of PD-L1 on tumor cell surfaces, enabling it to bind with PD-1 on immune cell surfaces, thereby suppressing immune cell activity [63]. Additionally, IL-18 induces tumor cells to secrete immunosuppressive cytokines such as IL-10 and TGF-β, further weakening the antitumor immune response [64]. This induction of immune evasion-related molecules indicates that IL-18’s role in tumor immune evasion extends beyond regulating immune cell functions to directly modulate tumor cell characteristics. IL-18BP can affect the expression levels of these immune-evasion-related molecules by regulating the effective concentration of IL-18.

#### 3.2.3. Interactions Between IL-18 and Other Cytokines and Signaling Pathways

The role of IL-18 in tumor immune evasion is not isolated but involves complex synergistic or antagonistic interactions with other cytokines and signaling pathways. First, the synergy between IL-18 and IL-12 holds significant importance in tumor immunity [65]. Studies have shown that the combined use of IL-18 and IL-12 can significantly enhance Th1-type immune responses and promote IFN-γ production [66]. This synergy not only helps activate CTL and NK, but also effectively inhibits tumor growth [67]. However, in certain situations, the effects of IL-18 may be counteracted by other cytokines. For example, IL-18BP secreted by tumor cells can competitively bind to IL-18, thereby weakening its antitumor activity [29]. These complex interactions indicate that the role of IL-18 in tumor immune evasion is regulated by various factors working together.

In summary, IL-18 exerts complicated and multifaceted roles in tumor immune evasion through interactions with other cytokines and signaling pathways. These interactions not only regulate immune cell functions but also directly influence the intrinsic characteristics of tumor cells, forming a sophisticated regulatory network. Future research should further explore the molecular mechanisms underlying these interactions to provide theoretical foundations for developing IL-18-based therapeutic strategies.

#### 3.2.4. Mechanism Analysis of IL-18’s Heterogeneity Effects

IL-18 exhibits heterogeneity in its effects across different tumor types, such as showing opposite effects in glioma and colorectal cancer. The underlying mechanism of this phenomenon deserves in-depth analysis [15,68]. From the perspective of tissue-specific interstitial interactions, the interstitial microenvironment of glioma is dominated by astrocytes and microglia, and IL-18 can activate microglia to release pro-inflammatory factors, enhancing local antitumor immunity [68]; while in colorectal cancer, the interstitial environment is rich in fibroblasts and intestinal microbiome derivatives, and IL-18 may induce fibroblasts to secrete MMPs, promoting tumor invasion [15,68]. From the analysis of cancer metabolic characteristics, glioma cells mainly rely on glycolysis, and the high metabolic level leads to lactate accumulation, which can enhance IL-18BP secretion and weaken the antitumor effect of IL-18 [21]; for some subtypes of colorectal cancer, they rely on fatty acid oxidation, and IL-18 can promote tumor cell survival by regulating lipid metabolism-related genes [21,62]. From the perspective of expression and regulatory patterns of IL-18BP, IL-18BP is mainly secreted by MDSCs in glioma tissues, while tumor cells in colorectal cancer can highly express IL-18BP themselves, resulting in varying degrees of inhibition of IL-18’s function [30,31]. Integrating the findings of existing controversial studies, the direction of IL-18’s effect may be determined by the ratio of “IL-18 activity/IL-18BP level”, with a high ratio tending to be antitumor and a low ratio promoting tumor progression. This hypothesis awaits further verification [8,63].

In conclusion, IL-18 plays complex and multifaceted roles in tumor immune evasion through interactions with other cytokines and signaling pathways. These interactions not only regulate the functions of immune cells but also directly affect the inherent characteristics of tumor cells, forming a complex regulatory network. Future research should further explore the molecular mechanisms of these interactions to provide a theoretical basis for developing treatment strategies based on IL-18 [64,66].

## 4. IL-18-Mediated Tumor Immune Evasion: Research Status and Targeted Therapeutic Strategies

### 4.1. Research Achievements Related to IL-18 and Tumor Immune Evasion

#### 4.1.1. Evidence from In Vitro and In Vivo Experimental Studies

The influence of IL-18 on the interaction between tumor cells and immune cells has been extensively studied. Research has shown that IL-18 can affect tumor immune evasion by regulating the activity of NK cells. In a mouse model of colon cancer transplanted tumors, IL-18 was proven to enhance the cytotoxicity of NK cells and promote tumor cell apoptosis by inducing the production of IFN-γ [68]. Further studies have indicated that IL-18, as a potential therapeutic target for boosting immunotherapy, also plays a role in regulating glioma cell response to chemotherapy. In glioma models and clinical tissue analyses, the high expression of IL-18 is closely associated with temozolomide chemoresistance, which is mainly mediated by activating the PI3K/AKT signaling pathway [68]. Additionally, IL-18 can also upregulate the expression of MMP-2, thereby enhancing the invasion and migration ability of tumor cells. It is worth noting that these effects show certain heterogeneity in different types of tumor cells. In glioma cells, the effect of IL-18 is mainly to inhibit tumor growth, while in colon cancer cells, it may have both promoting and inhibiting effects on tumors. In the mouse glioma model, bone marrow stromal stem cells transfected with IL-18 were found to significantly inhibit tumor growth and prolong the survival of the animals [69]. This result indicates that IL-18 may exert its antitumor effect by activating effector cells in the innate immune system, such as NK cells and macrophages. However, in mice without NK cells, the antitumor effect of IL-18 was significantly reduced, suggesting that NK cells play an important role in its mechanism of action [18]. Additionally, the role of IL-18 in tumor immune evasion is also subject to complicated regulation by the TME. In the K7M2 osteosarcoma model, IL-18 was found to be related to the recruitment of MDSCs, which are an important group of immunosuppressive cells in the TME [70,71]. Combination intervention of anti-PD-1 antibody and anti-IL-18 antibody, the research team observed an increase in the proportion of CD8+ T cells infiltrating the tumor and a reduction in tumor burden, indicating that IL-18 may promote tumor immune evasion by regulating the function of immunosuppressive cells [72].

Although significant progress has been made in the research of IL-18, its specific mechanism of action remains controversial. For instance, in certain tumor types, IL-18 may exhibit both protumor and antitumor effects simultaneously, which is closely related to its local concentration in the TME, receptor expression levels, and the interactions with other cytokines [29]. Therefore, further elucidation of the mechanism of IL-18’s action in different tumor models is of great significance for the development of IL-18-based therapeutic strategies.

#### 4.1.2. Clinical Research Evidence

In clinical research, the changes in IL-18 expression levels in different types of tumor patients and their correlations with tumor immune evasion and clinical prognosis have received extensive attention (Table 1). In the cancer tissues of patients with liver metastasis from colorectal cancer, the positive expression rate of IL-18 was significantly higher than that in normal adjacent tissues, and it was closely related to TNM stage, degree of differentiation, and lymph node metastasis [30]. Similarly, in the study of head and neck squamous cell carcinoma patients, the expression level of IL-18 was significantly negatively correlated with tumor TNM stage, lymph node metastasis, and patient survival, suggesting that it might serve as a potential biomarker for tumor prognosis [29]. Moreover, the expression of IL-18 in brain tumor patients also showed significant heterogeneity. The expression level of IL-18 was lower in well-differentiated tumor tissues and almost not expressed in poorly differentiated tumors, which might be related to the enhanced immune evasion ability of tumor cells [31]. It is worth noting that the expression level of IL-18 is not only related to the occurrence and development of tumors but also may affect the treatment response of patients. For example, in patients with hepatocellular carcinoma, changes in IL-18 levels are closely related to the efficacy of tumor necrosis factor alpha converting enzyme therapy, indicating that it may serve as an indicator for predicting treatment outcomes [73]. In breast cancer patients, IL-18 levels are closely associated with the activation of the PI3K/AKT signaling pathway. By inhibiting key molecules in this pathway, IL-18 expression can be indirectly reduced [74].

It should be noted that the phenomenon of “IL-18 is expressed at a lower level in well-differentiated tumors and is almost not expressed in poorly differentiated tumors” is not a universal rule, but rather a specific manifestation of certain tumor types (such as brain tumors, some solid tumors) [31,73]. The potential impact of this expression pattern on tumor immune evasion is as follows: poorly differentiated tumor cells reduce the activation of NK cells and CD8+ T cells by low expression of IL-18, and also reduce the pro-inflammatory response mediated by IL-18, thereby evading immune surveillance; in addition, low IL-18 expression may be related to the EMT process of tumor cells, further enhancing their invasive and metastatic abilities [62]. Therefore, the IL-18 expression pattern can be used as a prognostic marker for some tumors, providing a reference for clinical assessment [31,73].

### 4.2. Therapeutic Strategies and Targets for IL-18-Mediated Tumor Immune Evasion

#### 4.2.1. Potential Therapeutic Strategies

Given IL-18’s involvement in cancer immune evasion, different strategies were explored to downregulate its actions. IL-18 signaling can be blocked using antibodies or small molecules. Anti-IL-18 antibodies can compete with IL-18 for binding to IL-18R, thereby decreasing its regulatory effect on immune cells. IL-18 activates the myd88-induced signaling pathway, which in turn activates the NF-κB and MAPK pathways, leading to the release of pro-inflammatory cytokines [75]. Therefore, using small molecule inhibitors targeting key components of MyD88 or NF-κB can effectively inhibit the immune evasion induced by IL-18. In the K7M2 osteosarcoma model, IL-18 is associated with the recruitment of MDSCs [71]. Combining the use of IL-18 inhibitors can significantly increase CD8+ T cell infiltration and reduce tumor burden [62]. These findings indicate that blocking the IL-18 signal not only directly weakens the immunosuppression in the TME but also enhances the efficacy of other immunotherapies. In the field of CAR-T cell therapy, IL-18 is widely used to enhance the antitumor activity of CAR-T cells. The review in *The International Journal of Molecular Science* in 2024 includes multiple related studies [19]. Hu B. et al. [76] developed IL-18-secreting CAR-T cells, which significantly improved the therapeutic efficacy against solid tumors by enhancing T cell proliferation, survival, and cytotoxicity. These CAR-T cells can continuously secrete IL-18 within the TME, effectively overcoming immune suppression and augmenting the persistence of antitumor immune responses. However, clinical application of CAR-T cell therapy is confronted with challenges including cytokine release syndrome (CRS), neurotoxicity, and hemophagocytic lymphohistiocytosis. Walton, Z.E. et al. systematically summarized existing and emerging pharmacotherapeutic strategies for these adverse reactions: for CRS, first-line treatments primarily include IL-6 receptor antagonist tocilizumab and glucocorticoids, which can potently suppress overactivated immune responses; for neurotoxicity, current management mainly relies on supportive care and symptomatic interventions, while novel targeted agents such as blood–brain barrier-penetrating IL-1 receptor antagonists are under development [77]. These findings provide crucial evidence for the safe translation of IL-18-related CAR-T cell therapy into clinical practice.

Modulating IL-18 expression levels represents another potential therapeutic strategy in the TME. Gene therapy and pharmacological interventions are two primary approaches. In gene therapy, technologies like CRISPR-Cas9 can knock out or silence IL-18 genes, thereby reducing their production at the source and inhibiting their role in promoting tumor immune evasion. Additionally, RNA interference technology specifically degrades IL-18 mRNA, effectively lowering its expression levels. Regarding pharmacological interventions, certain compounds have been shown to regulate IL-18 expression. In breast cancer patients, IL-18 levels are closely associated with the activation of the PI3K/AKT signaling pathway. By inhibiting key molecules in this pathway, IL-18 expression can be indirectly reduced [74]. It is worth noting that although these methods have shown potential in in vitro experiments and animal models, their clinical application still requires further verification, especially in terms of safety and efficacy. Due to the pleiotropic functions of IL-18, inhibiting IL-18 may cause side effects, such as weakening the body’s immune defense against pathogens and inducing autoimmune diseases, etc. A balance between efficacy and risk needs to be achieved in the treatment [54].

#### 4.2.2. Analysis of Therapeutic Targets

IL-18R and its associated signaling molecules demonstrate significant therapeutic potential as therapeutic targets. Composed of two subunits, IL-18Rα binds to IL-18 while IL-18Rβ initiates signaling pathways. Antibodies or small molecule inhibitors targeting IL-18Rα can effectively block IL-18R binding, thereby inhibiting downstream signaling activation. Additionally, MyD88, a core molecule in IL-18 signaling, phosphorylates IL-1 receptor-associated kinase upon binding to IL-18R, recruits TRAF6, and ultimately activates NF-κB and MAPK pathways. Consequently, MyD88 has emerged as a promising therapeutic target. Studies indicate that inhibiting MyD88 expression or activity significantly reduces IL-18-induced pro-inflammatory cytokine release [78], thereby mitigating immune suppression in TME. Furthermore, small molecule inhibitors targeting TRAF6 or IRAK have shown promising antitumor potential, particularly when combined with other immunotherapies to synergistically enhance therapeutic efficacy.

#### 4.2.3. Advances and Challenges in Therapeutic Strategies in Basic and Clinical Research

In the field of basic research, therapeutic strategies targeting IL-18-mediated tumor immune evasion have made significant progress (Table 1). Experiments have shown that using anti-IL-18 antibodies or small molecule inhibitors to block IL-18 signal transduction can significantly inhibit the proliferation and invasiveness of tumor cells. The use of IL-18BP significantly reduced the infiltration rate of MDSC in tumor tissues and enhanced the activity of CD8+ T cells [8,71]. The combination of anti-PD-1 antibody and anti-IL-18BP antibody or IL-18 mutant significantly increased the proportion of CD8+, IFN-γ positive, and granzyme B positive T cells in tumors, effectively inhibiting tumor growth [8]. These findings indicate that blocking IL-18 signal transduction not only directly weakens the immunosuppressive effect in the TME but may also enhance the efficacy of other immunotherapeutic approaches. Moreover, gene therapy and RNA interference techniques have shown great potential in basic research. Using CRISPR-Cas9 technology to knockout the IL-18 gene significantly reduced the proliferation and invasiveness of tumor cells and inhibited tumor angiogenesis [79].

**Figure 3:**
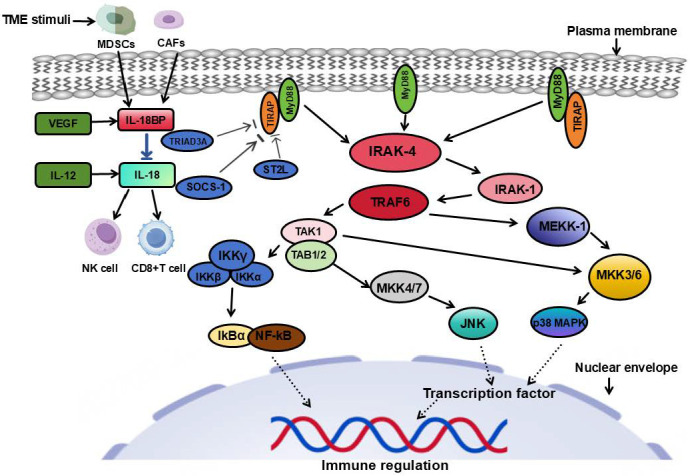
The signaling network diagram of the IL-18/IL-18BP axis in the TME.

Although significant progress has been made in basic research, the therapeutic strategies for IL-18 still face numerous challenges in clinical trials. Firstly, the clinical trial results of anti-IL-18 antibodies indicate that although they can partially inhibit tumor growth, the clinical application of anti-IL-18 antibodies is still in the early exploration stage, with limited relevant clinical data and no clear therapeutic conclusions have been formed yet. Some patients experience severe immune-related adverse reactions. Secondly, small molecule inhibitors targeting downstream signaling molecules of IL-18 show certain toxic side effects, especially causing significant damage to normal tissues. Moreover, although gene therapy and RNA interference technologies have shown certain potential in small-scale clinical trials, their safety and long-term efficacy still require further verification [80]. Studies have confirmed that drug intervention to reduce IL-18 expression significantly improves the prognosis of patients, but some patients experience liver dysfunction and other side effects [81]. Therefore, future research should focus more on optimizing treatment strategies—especially achieving precise regulation—to maximize efficacy and minimize side effects. At the same time, combining these therapies with other immunotherapies may become a key direction for breaking through the limitations of single therapy. As shown in Figure 3, the diagram depicts the signaling network of the IL-18/IL-18BP axis in the TME and identifies key pathways amenable to therapeutic intervention.

### 4.3. Immunotoxicity and Corresponding Management Strategies

IL-18 exhibits potent pro-inflammatory activity, which poses significant immunotoxicity risks to its clinical application [82]. Both the administration of exogenous IL-18 and IL-18-secreting CAR-T cell therapy may disrupt the systemic immune homeostasis of the organism, leading to severe adverse events primarily including CRS and neurotoxicity [83,84] (Table 2). This paper also elaborates on key translational risks such as macrophage activation syndrome (MAS) and NK cell hyperactivation to improve the safety assessment system for IL-18-related therapies [82,85].

#### 4.3.1. Cytokine Release Syndrome (CRS)

Excessive systemic IL-18 triggers the cascade release of pro-inflammatory cytokines (e.g., IL-6, TNF-α) through the NF-κB and MAPK signaling pathways, ultimately inducing CRS [82]. Its clinical manifestations range from mild symptoms such as fever and myalgia to severe, life-threatening conditions including refractory hypotension, capillary leak syndrome and multiple organ failure [83]. Notably, IL-18-secreting CAR-T cells carry a significantly higher risk of CRS compared with conventional CAR-T products, due to their sustained secretion of IL-18 in the TME that continuously amplifies the inflammatory cascade [84]. For the clinical management of CRS, close vital sign monitoring and symptomatic supportive care are sufficient for mild cases; in contrast, immediate combined pharmacological intervention is required for severe grade ≥3 CRS, including IL-6 receptor antagonists (e.g., tocilizumab) and low to moderate doses of glucocorticoids, to rapidly block the inflammatory cascade and prevent disease progression.

#### 4.3.2. Neurotoxicity

IL-18-induced neurotoxicity in CAR-T therapy is primarily associated with impaired integrity of the blood–brain barrier (BBB) [86]. IL-18 can activate vascular endothelial cells of the BBB, upregulate the expression of vascular adhesion molecules and increase BBB permeability, thereby allowing peripheral pro-inflammatory factors (e.g., IL-6, IFN-γ) and activated immune cells to infiltrate the central nervous system (CNS) and further induce neuroinflammation [82,86]. The clinical symptoms range from mild headache and mild cognitive decline to severe manifestations such as seizures and cerebral edema [86]. At present, there are no specific therapeutic agents for this type of neurotoxicity, and clinical practice focuses on prevention and early intervention: real-time monitoring of neurological symptoms during treatment, and prompt symptomatic treatments such as dehydration for intracranial pressure reduction and anticonvulsant therapy once abnormal symptoms occur [86].

#### 4.3.3. Macrophage Activation Syndrome (MAS)

Sustained and excessive IL-18 stimulation is an important factor inducing MAS in IL-18-related immunotherapies [1]. IL-18 can directly activate tissue-resident macrophages and monocyte-derived macrophages in multiple organs, leading to uncontrolled macrophage activation and massive release of pro-inflammatory cytokines and cytotoxic molecules, which in turn trigger systemic hyperinflammation and multi-organ damage [82,84]. The clinical manifestations include persistent high fever, hepatosplenomegaly, cytopenia, and abnormal liver function [84]. IL-1 receptor antagonists (e.g., anakinra) are the first-line choice for clinical immunosuppressive therapy, which can specifically block the IL-18/IL-1 signaling pathway and inhibit excessive macrophage activation; glucocorticoids can be used as adjuvant therapy for severe cases [82].

#### 4.3.4. NK Cell Hyperactivation

IL-18 is a key cytokine regulating the activation and cytotoxicity of NK cells [85]. Although enhanced NK cell activity can synergistically improve antitumor immune efficacy [1], uncontrolled NK cell activation induced by sustained IL-18 secretion may cause off-target immune damage to normal tissues and even trigger autoimmune reactions. The clinical manifestations include unexplained tissue damage, abnormal liver and kidney function, and autoimmune hemolytic anemia [85]. It is recommended to monitor the activation status of peripheral blood NK cells (e.g., the expression of CD69 and CD107a) during clinical treatment [85]; for patients with obvious NK cell hyperactivation, low-dose selective NK cell inhibitors or short-course low-dose glucocorticoids can be used as appropriate to regulate cell activity and reduce off-target immune damage.

## 5. Conclusions and Future Perspectives

### 5.1. Precise Regulation of IL-18 to Block Tumor Immune Evasion

With the continuous advancement of precision medicine concepts, achieving precise regulation of IL-18 through gene editing technology has become one of the key research directions in the future. As a multifunctional cytokine, IL-18 plays a complex and diverse role in tumor immune evasion, making its precise intervention dependent on highly specific technical approaches. For instance, the CRISPR-Cas9 system can target-modify the IL-18 gene or its receptor coding sequence to inhibit its expression or function at the transcriptional level [79]. Additionally, RNA interference technology has been widely applied to regulate key molecules in the IL-18 signaling pathway, to block downstream signal transmission and weaken tumor cells’ immune evasion capabilities [87]. However, practical applications of these technologies still face challenges including off-target effects, immunogenicity, and long-term safety concerns. Future research should focus on optimizing these technological platforms while integrating single-cell sequencing and multi-omics analysis to deeply explore the dynamic expression patterns and regulatory networks of IL-18 in different TME, providing theoretical support for developing more precise therapeutic strategies.

Meanwhile, systems biology and computational immunology methods have provided key technical support for analyzing the IL-18 regulatory network and achieving precise regulation [88]. Empirical studies have confirmed that by integrating multi-omics data (transcriptome, proteome, cytokine profile), and using bioinformatics algorithms, an interaction network between IL-18 and components of the TME (immune cells, cytokines, signaling pathways) can be constructed, enabling precise identification of the core molecular nodes regulating IL-18-mediated immune evasion [83]. For instance, module analysis based on machine learning has successfully screened out key gene clusters related to IL-18, which not only can reflect the differences in patients’ immune status but also can provide data support for the selection of target treatment targets [83]. In the future, with the deep integration of single-cell sequencing, spatial transcriptomics, and computational models, it is expected to further reveal the dynamic regulatory patterns of IL-18 in different TMEs, providing a more solid theoretical basis for the development of precise regulatory strategies.

### 5.2. The Role of IL-18 in Emerging Tumor Therapy Models

In recent years, the emergence of innovative cancer treatment models combining immunotherapy with chemotherapy and radiotherapy has opened new frontiers for IL-18 research. As a crucial bridge connecting innate immunity and adaptive immunity, IL-18’s role in combination therapies has garnered increasing attention. On one hand, IL-18 enhances ICIs efficacy by boosting NK cell and T cell activity. Research has shown that the combination of anti-PD-1 antibodies with IL-18BP inhibitors or bait resistant IL-18 variants can significantly increase the infiltration of CD8^+^ T cells in tumor tissues, while effectively reducing tumor burden [18,89]. On the other hand, IL-18 may exert synergistic effects during radiotherapy and chemotherapy. Studies indicate that radiation-induced tumor cell apoptosis releases large amounts of DAMPs, which activate inflammatory bodies and promote IL-18 secretion, thereby amplifying antitumor immune responses [22].

However, the specific mechanisms of IL-18 in combination therapy still require further elucidation. How does IL-18 synergize with other cytokines (such as IL-12 and IL-2) to optimize therapeutic outcomes? Is there an interaction between its signaling pathways and cellular stress responses induced by chemotherapy drugs or radiation therapy? Addressing these questions will help reveal the potential value of IL-18 in combination treatment. Additionally, it is noteworthy that IL-18’s dual role—both enhancing antitumor immunity and potentially promoting tumor progression—requires careful balancing in combination therapies. Therefore, future research should focus on how to maximize IL-18’s antitumor effects while avoiding its potential carcinogenic risks, thereby achieving better therapeutic outcomes.

### 5.3. IL-18 Threshold and Contextual Regulation Model

Based on the existing research results, this review proposes the “IL-18 Threshold and Contextual Regulation Model” to integrate key factors such as concentration, receptor expression, cytokines, and components of the TME (Figure 3) [8,17,63]. This model suggests that the immune stimulating/inhibiting effect of IL-18 is jointly determined by “threshold” and “context”: when the effective concentration of IL-18 (the ratio of free IL-18 to IL-18BP) exceeds a specific threshold, and there are co-stimulatory cytokines such as IL-12 and high expression of IL-18R in the TME, IL-18 tends to activate antitumor immunity [17,64]; when the effective concentration of IL-18 is below the threshold, or when immune suppressive cells are enriched in the TME and VEGF and other tumor-promoting factors are highly expressed, IL-18 promotes tumor immune escape [8,15].

This model can explain the heterogeneity effect of IL-18 in different tumors: in glioma, the secretion of IL-18BP is relatively low, and the expression level of IL-12 is high, so the effective concentration of IL-18 is likely to exceed the threshold, and thus mainly shows an antitumor effect [68]; in colorectal cancer, tumor cells themselves highly express IL-18BP, and tumor-promoting factors such as VEGF are enriched, so the effective concentration of IL-18 is below the threshold, and thus tends to promote tumor progression [15,30].

### 5.4. Future Research Directions

Based on the above mechanistic analysis of the IL-18/IL-18BP axis in tumor immune escape and the established “IL-18 threshold and context-dependent regulatory model”, future research should focus on three core aspects: verification of key mechanisms, development of detection technologies, and optimization of clinical translational strategies [90]. The aim is to achieve precise regulation of IL-18 function and promote the clinical translation of IL-18-based immunotherapies, with the specific research priorities as follows:

Further verify the tumor-specific threshold of functional IL-18 activity and the regulatory mechanism of IL-18BP-mediated dominant inhibition. Construct organoid models and humanized tumor xenograft models of different malignant tumors such as colorectal cancer, glioma and esophageal cancer to clarify the dynamic variation of the IL-18/IL-18BP ratio in the TME and its regulatory effect on antitumor immune responses. Further explore the molecular mechanism underlying the IL-18BP-mediated dominant inhibition of decoy-resistant IL-18 variants and identify the key binding domains between IL-18BP and IL-18 [91], so as to provide a theoretical basis for the design of highly effective and low-toxic IL-18BP-targeted antagonists.

Develop TME-specific detection technologies for functional IL-18 activity and IL-18BP expression. Current detection methods mostly focus on the total IL-18 level in peripheral blood or tumor tissues, which cannot accurately reflect the actual bioavailability of IL-18 in the TME. Future research needs to develop minimally invasive detection tools such as specific antibodies and molecular probes to achieve real-time and accurate quantification of the free IL-18/IL-18BP ratio in the TME at the single-cell level [92]. Meanwhile, combine multi-omics and machine learning technologies to establish an IL-18-based immunotherapy efficacy prediction model with the IL-18/IL-18BP ratio, tumor type and immune microenvironment characteristics as indicators, so as to realize precise stratification of patients suitable for IL-18-targeted therapy.

Optimize the design of targeted therapeutic strategies for the IL-18/IL-18BP axis and explore efficient combination therapy regimens. On the one hand, develop TME-responsive IL-18BP antagonists by using technologies such as antibody-drug conjugation (ADC) and tumor-specific protease cleavage, to achieve targeted activation of IL-18’s antitumor activity in the TME and reduce systemic immune side effects caused by non-specific activation of IL-18. On the other hand, based on the synergistic effects of IL-18 with other immune checkpoints and cytokines, explore combination therapy regimens of IL-18BP antagonists with PD-1/PD-L1 inhibitors, IL-12 agonists or chemoradiotherapy, so as to enhance antitumor immune responses through multiple pathways and reverse the immunosuppressive state of the TME [92,93]. In addition, develop local delivery systems (e.g., sustained-release microspheres and oncolytic viruses) for IL-18 agonists to effectively increase the local concentration of IL-18 in tumor tissues and avoid the occurrence of CRS.

Conduct large-sample prospective clinical trials to evaluate the efficacy and safety of IL-18/IL-18BP axis-targeted therapies [92]. At present, most IL-18-related therapeutic strategies are still in the preclinical or early clinical trial stage, and their clinical efficacy and safety need further verification. Future clinical research should strictly follow the principle of patient stratification, screen eligible subjects according to the IL-18/IL-18BP ratio and TME immune characteristics, and systematically evaluate the short-term and long-term efficacy of targeted therapies, as well as the incidence of adverse reactions such as CRS and autoimmune diseases, so as to provide evidence-based medical evidence for the standardized clinical application of IL-18/IL-18BP axis-targeted therapies.

## Figures and Tables

**Table 1:** IL-18 Expression and clinical treatment methods of different tumor types.

Type of Tumor	IL-18 Expression Level	Association with Immune Evasion	Therapeutic Method
Colorectal cancer	Highly expressed in cancer	The degree of CD8^+^ T cell infiltration was reduced	Anti-IL-18BP agents alone or in combination with immunotherapy
Liver cancer	Highly expressed in cancer	Induce MDSCs aggregation and inhibit the cytotoxic function of NK cells	Attempt to arm CAR-T cells with IL-18 to target liver cancer cells
head and neck squamous cell carcinoma	Highly expressed in cancer	Suppress the antitumor activity of CD4^+^ T cells Evaluate	IL-18 agonists combined with PD-L1 inhibitors
Gastric cancer	Highly expressed in cancer	Promote VEGF secretion and weaken antitumor immune response	IL-18-related immune markers guide chemotherapy combined with immunotherapy
Triple-negative breast cancer	Significantly elevated in cancer tissues	Promote the polarization of TAM to the M2 type and construct an immunosuppressive microenvironment	Anti-IL-18BP drugs, either used alone or in combination with immunotherapy
Non-small cell lung cancer	Significantly elevated in cancer tissues	Inhibitory effect on T cell activity	Exploring anti-IL-18BP drugs combined with PD-1 inhibitors

**Table 2:** Main types of immune toxicity, pathogenic mechanisms and clinical management strategies of IL-18-related therapies.

Type of Immune Toxicity	Core Pathogenic Mechanism Main Clinical	Manifestations	Clinical Management Strategy
Cytokine Release Syndrome (CRS)	IL-18 will trigger the release of a series of pro-inflammatory cytokines (such as IL-6 and TNF-α) through the NF-κB/MAPK pathway.	Mild cases: fever, myalgia, fatigue; Severe cases:	IL-6 receptor antagonist (tocilizumab) + low to moderate dose glucocorticoids
Neurotoxicity	IL-18 can activate the vascular endothelial cells of the blood–brain barrier, upregulate the expression of adhesion molecules, and trigger neuroinflammation.	Mild cases: headache, dizziness, mild cognitive decline;	Core: prevention + early intervention; real-time monitoring of neurological symptoms, timely symptomatic treatments such as dehydration to reduce intracranial pressure and anti-convulsion when abnormalities occur
Macrophage Activation Syndrome (MAS)	Excessive IL-18 can activate multiple macrophages, causing them to become uncontrollably activated and release large amounts of pro-inflammatory cytokines, thereby triggering systemic excessive inflammation and multiple organ damage.	Persistent high fever, enlarged liver and spleen, elevated inflammatory markers, and abnormal liver function.	First-line: IL-1 receptor antagonist (anakinra) to block IL-18/IL-1 signaling; Severe cases: combined with glucocorticoids for adjuvant immunosuppression
NK cell overactivation	IL-18 can enhance the activity and toxicity of NK cells, thereby triggering non-specific immune attacks on normal tissues.	Persistent high fever, enlarged liver and spleen, elevated inflammatory markers, and abnormal liver function.	low-dose selective NK cell inhibitors/short-course low-dose glucocorticoids

## Data Availability

No new data were created or analyzed in this study. Data sharing is not applicable to this article.

## Abbreviations

The following abbreviations are used in this manuscript:

AP-1	Activated protein-1
ADCC	Antibody-dependent cell-mediated cytotoxicity
CAR-T	Chimeric antigen receptor T-cell
CTL	Cytotoxic T cells
CTLA-4	Cytotoxic T lymphocyte-associated antigen-4
CRS	Cytokine release syndrome
DC	Dendritic cells
DAMPs	Damage-associated molecular patterns
EMT	Epithelial-mesenchymal transition
GM-CSF	Granulocyte-macrophage colony-stimulating factor
GZMB	Granzyme B
PRF1	Perforin 1
IFNG	Interferon gamma
IL-2	Interleukin-2
IRAK1	Interleukin-1 receptor-associated kinase 1
CAFs	Cancer-associated fibroblasts
ST2L	Soluble ST2 (long form)
MAPK	Mitogen-activated protein kinase
TAK1	Transforming growth factor-β-activated kinase 1
CD8^+^ T cell	CD8-positive T cell
IRF	Interferon regulatory factor
IL-12	Interleukin-12
TRAP	TNF receptor-associated protein
RIP	Receptor-interacting protein
IL-18	Interleukin-18
IL-18Rs	IL-18 receptors
IL-18BP	IL-18-binding proteins
ICIs	Immune checkpoint inhibitors
IFN-γ	Interferon-γ
PBMC	Peripheral blood mononuclear cell
JNK	c-Jun endonucleotide-binding kinase
MDSCs	Myeloid-derived suppressor cells
MHC	Major histocompatibility complex
MMP-2	Matrix metalloproteinase-2
MMPs	Matrix metalloprMatrix metalloproteinasesoteinases
MyD88	Myeloid differentiation factor 88
NK	Natural killer
NF-κB	Nuclear factor-κB
PD-1	Programmed death receptor-1
IRAK-4	Interleukin-1 Receptor-Associated Kinase
IRAK-1	TRIF-related adaptor molecule
TRAM	Toll/Interleukin-1 Receptor domain
TIR	IκB Kinase
IKKα	IκB Kinase Alpha
IKKβ	IκB Kinase Beta
TAB1/2	TAK1 Binding Protein 1/2
IKKγ/NEMO	IκB Kinase Gamma
IκBα	Inhibitor of Nuclear Factor κB Alpha
PI3K	Phosphatidylinositol 3-kidronase
PD-L1	Programmed cell death ligand-1
PAMPs	Pathogen-associated molecular patterns
TRAF-6	TNF-α receptor-associated factor-6
TME	The tumor microenvironment
TAMs	Tumor-associated macrophages
TGF-β	Transforming growth factor-β
Tregs	Regulatory T cells
VEGF	Vascular endothelial growth factor
